# GNSS Spoofing Detection via the Intersection Angle between Two Directions of Arrival in a Single Rotating Antenna

**DOI:** 10.3390/s24041116

**Published:** 2024-02-08

**Authors:** Shimiao Chen, Shuyan Ni, Tuofeng Lei, Lingfeng Cheng, Xin Song

**Affiliations:** 1 Space Engineering University, Beijing 101416, China; 14291002@bjtu.edu.cn (S.C.);; 2 China Satellite Maritime Telemetry Control Department, Jiangyin 214400, China; 3 Academy of Military Sciences, Beijing 100071, China

**Keywords:** GNSS spoofing detection, antenna, GLRT, direction of arrival, intersection angle

## Abstract

Spoofing against the Global Navigation Satellite System (GNSS) is an attack with strong concealment, posing a significant threat to the security of the GNSS. Many strategies have been developed to prevent such attacks, but current detection methods based on signal direction for multi-agent spoofing require multiple antennas/receivers, leading to increased cost and complexity in implementation. Additionally, methods utilizing a moving single antenna cannot effectively detect multi-agent spoofing. Therefore, we introduce a novel spoofing-detection technique based on the intersection angle between two directions of arrival (IA-DOA) using a single rotating antenna. The essence of this approach lies in estimating the IA-DOA between a pair of signals by utilizing the carrier-to-noise ratio (CNR) and carrier phase single difference (CPSD) of the received signal. The estimation of IA-DOA should be consistent with the prediction when there is no spoofing. With spoofing, it is difficult to accurately simulate the directionality of navigation signals, which can disrupt the consistency between the estimation and prediction of IA-DOA. Therefore, estimations and predictions of IA-DOA can be used to establish detection variables through generalized likelihood ratio testing (GLRT) to detect multi-agent spoofing. We conducted a simulation to analyze the impact of the antenna’s parameters on the detection performance and evaluated it through on-site experiments. The results indicate that the method proposed in this article can efficiently achieve real-time detection of multi-agent spoofing.

## 1. Introduction

The application of the Global Navigation Satellite System (GNSS) in military and civilian fields is gradually becoming widespread, and society’s dependence on PNT (positioning, navigation, and timing) services provided by GNSS is rapidly increasing. At this time, the safety and stability of the GNSS have also received great attention. Due its weak signal strength, open signal modulation methods, and predictable partial navigation data, the GNSS is highly susceptible to spoofing [1,2], which is fatal to navigation systems. Therefore, the detection of spoofing is very important.

Currently, many spoofing methods have been proposed. In the early stages of research on spoofing-detection methods, scholars conducted spoofing detection by searching for signal features that changed after a single antenna receiver was spoofed. Due to the inability of spoofing to perfectly simulate the power of real signals, the addition of spoofing can cause changes in the signal power [3], the carrier-to-noise ratio (CNR) [4], etc. In the acquisition and tracking stage, under the covert spoofing strategy, the spoofing needs to enter the tracking loop, slowly increase the power, and change the phase, which can cause misalignment between the real code and the spoofing code phases and the appearance of multiple correlation peaks [5]. Based on the changed signal features mentioned above, spoofing detection can be performed, which has good detection performance for simple spoofing and is currently the most widely used spoofing-detection algorithm. The combination of inertial navigation and GNSS can achieve spoofing detection by conducting consistency checks on features such as trajectory, acceleration, and position. Broumandan et al. [6] compared the trajectory estimated by a GNSS receiver with the trajectory obtained by the INS. Kwon et al. [7] compared the accelerations calculated using accelerometers and GNSS receivers. Currently, many scholars use encryption and authentication techniques in cryptography to encrypt civilian navigation signals, making it difficult to predict the navigation signals. Moreover, receivers can judge the integrity of the received signals, thereby better resisting spoofing attacks. According to the different encryption-authentication methods and objects, this method can be divided into navigation message authentication (NMA) [8,9] and spreading code authentication (SCA) [10]. The spoofing-detection method based on signal encryption authentication requires modifying the navigation signal system, which is difficult to achieve in the short term.

In addition, many scholars detect spoofing based on the direction differences between spoofed signals and real signals and other variables caused by different directions. Zhang et al. [11] proposed a method for the spoofing detection and suppression of pre-spread signals using array antennas. This method utilizes the original baseband signal to establish a cyclic correlation matrix, perform singular value decomposition, and perform a cyclic correlation eigenvalue test (CCET) to determine whether there is spoofing. If there is spoofing, the feature vector is used to establish a shadow space to eliminate spoofing. He et al. [12] proposed a spoofing-detection technology based on dual antennas. The essence of this method is to accurately estimate the frequency difference of arrival between a pair of fixed antennas based on carrier phase observation and navigation information. When there is no spoofing, the observations should be consistent with the predictions. Otherwise, due to the geometric and kinematic differences between the GNSS satellite and the spoofer, the spoofing will disrupt consistency, so multi-agent spoofing can be detected. Chen et al. [13] used the position information of multiple antennas to derive the intersection angle between two directions of arrival (IA-DOA) of different signals. Based on the predicted and estimated values of IA-DOA, generalized likelihood ratio testing (GLRT) was performed to achieve multi-direction spoofing detection. Seo et al. [14] proposed a spoofing-detection method using the norm of the difference of baseline vectors (NDB) of multiple receivers. This method has a low probability of fault detection and fast response time and can achieve instant anti-spoofing. The above methods require array antennas or multiple receivers, which is costly. Therefore, scholars proposed using a moving single antenna for signal direction estimation. Li et al. [15] proposed a spoofing-detection method based on a reciprocating antenna, which determines the direction of the signal by determining the relationship between the amplitude change caused by the antenna’s up and down motion and the signal incidence angle, achieving spoofing detection. In addition, based on the architecture of a single rotating antenna, similarity testing can be performed on the changing trends of carrier phases [16], CNR [17], and power [18] under the single rotating antenna to determine whether the navigation signal direction is consistent, thus achieving spoofing detection. However, the spoofing-detection method based on a moving single antenna cannot detect spoofing from multiple directions, and achieving spoofing detection from multiple directions still requires multiple antennas or receivers. The achievement of multi-agent spoofing detection based on a moving single antenna remains a challenge.

Therefore, this article applies the IA-DOA of two signals to a single rotating antenna and proposes a detection method for multi-agent spoofing. It is difficult for multi-agent spoofers to accurately simulate the directionality of navigation signals, which means that there may be deviations between the incident direction of the spoofing and the real signal. Based on the CNR and carrier phase single difference (CPSD) of the signal obtained from a single rotating antenna, the maximum likelihood estimation (MLE) is used to obtain the estimated values of IA-DOA for any two signals. The predicted values of IA-DOA are calculated using ephemeris information, and detection variables are established based on the predicted and estimated values to determine whether there is spoofing in the two signals. Performing GLRT testing on each set of signals can achieve spoofing detection. We conduct a detailed analysis of the parameters that affect detection performance through simulation. In addition, the effectiveness of the proposed method was verified and evaluated through on-site experiments. The method proposed in this article simultaneously utilizes the CNR and CPSD of a single rotating antenna to estimate IA-DOA, achieving real-time detection of multi-agent spoofing while improving the detection performance. It should be noted that in this method, the antenna is in motion, but the center of the motion trajectory should remain unchanged. The method proposed is only applicable to spoofing detection at fixed sites.

The detailed derivation of the principles and theories is conducted in Section 2. The spoofing-detection method based on the IA-DOA with a single rotating antenna is proposed and a detailed analysis of the feature parameters that affect detection performance through simulation is conducted in Section 3. Section 4 validates and evaluates the proposed method based on the on-site experiments. Section 5 concludes with discussions.

## 2. System Model

This section reveals the variation laws of the CNR and CPSD in a single rotating antenna and proposes a method for estimating the signal arrival angle difference using CNR and CPSD.

### 2.1. Single Rotating Antenna Model

**Figure 1:**
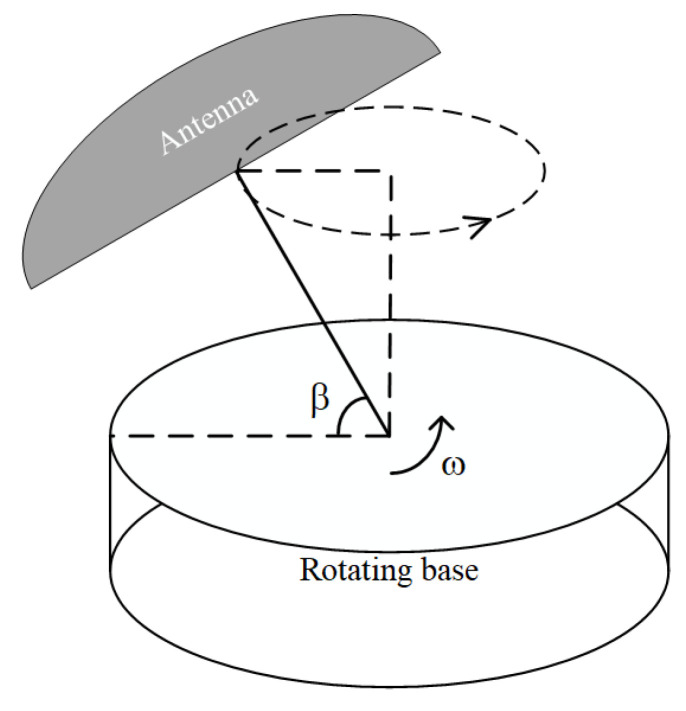
Schematic diagram of a single rotating antenna model.

The single rotating antenna model used in this article is shown in Figure 1. The antenna is fixed at an angle β to the rotating base, which rotates at an angular velocity ω [16].

**Figure 2:**
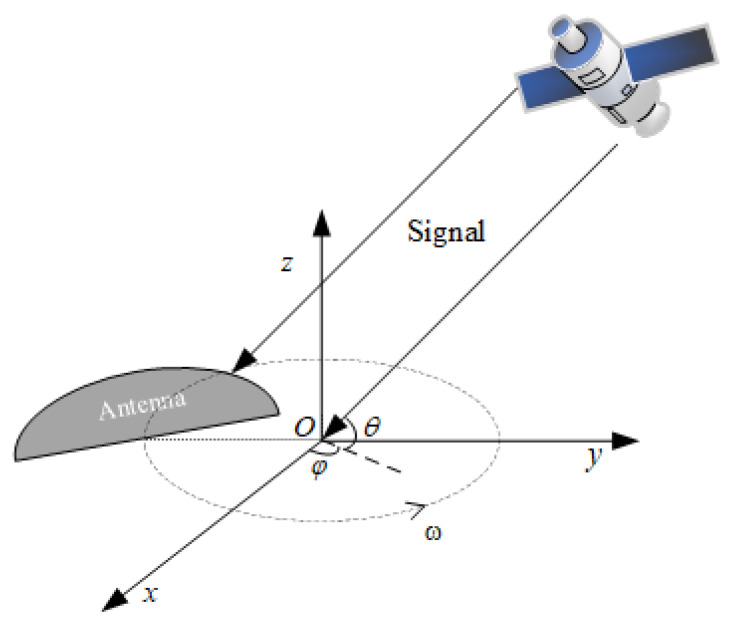
Rotating antenna coordinate system.

Due to the rotational motion of the antenna, the position of the antenna changes regularly, and the carrier phase of the same navigation satellite signal received by the receiver also changes regularly. During the antenna rotation process, the incident direction of the navigation signal changes in transmission, which leads to a regular change in the gain of the received signal. Their variation patterns are the same for different spoofing signals from the same direction, while their variation patterns are different for real signals from different directions. Therefore, spoofing detection can be carried out based on the above characteristics. First, there is a requirement to establish a Cartesian coordinate system with the center of the rotating circle at the center of the antenna as the origin of the coordinate system. The axis points toward the direction from the origin to the antenna’s phase center at time 0, as shown in Figure 2. The following will provide a detailed introduction to the changes in signal characteristics caused by rotating the antenna.

### 2.2. Parameter Estimation Based on CNR

The receiving antenna has different radiation and reception capabilities in different directions and has different signal gains for signals with varying angles of the incident. First, the changes in antenna gain during the rotation process of the antenna are analyzed. There is a requirement to establish a station center coordinate system with the antenna center as the origin, with the Z-axis pointing towards the zenith, the Y-axis pointing north, and the X-axis pointing east. The unit vectors corresponding to the antenna axis direction and signal direction are:

(1) $$\begin{array}{c} A={\left[\cos\left(\beta \right)\cos\left(\varphi_{0}-\omega t\right),\cos\left(\beta \right)\sin\left(\varphi_{0}-\omega t\right),\sin\left(\beta \right)\right]}^{T}\\ B={\left[\cos\left(\theta \right)\cos\left(\varphi \right),\cos\left(\theta \right)\sin\left(\varphi \right),\sin\left(\theta \right)\right]}^{T} \end{array}$$

where β is the elevation angle of the antenna axis, $\varphi_{0}$ is the initial azimuth angle of the antenna axis, *t* is the rotation time, θ is the signal incident elevation angle, and φ is the signal incident azimuth angle.

Assuming that the gain direction of the antenna is consistent, the antenna’s gain to the incident signal is mainly affected by the angle between the two unit vectors. The antenna gain can be expressed as [18]:

(2) $$G_{R}=f\left(\hat{\theta }\right)=f\left[\frac{\pi }{2}-\arccos\left(A\cdot B\right)\right]=f\left[\frac{\pi }{2}-\arccos\left(\cos\left(\theta \right)\cos\left(\beta \right)\cos\left(\varphi -\varphi_{0}+\omega t\right)+\sin\left(\beta \right)\sin\left(\theta \right)\right)\right]$$

where $\hat{\theta }$ represents the elevation angle of the signal direction relative to the antenna’s normal plane, indicating that the antenna gain is affected by the direction of the incident signal, the tilt angle of the rotating antenna, the speed of rotation, and time. Therefore, the antenna gain can be expressed as the function $G_{R}=f\left(\varphi ,\theta ,\beta ,\omega ,t\right)$.

**Figure 3:**
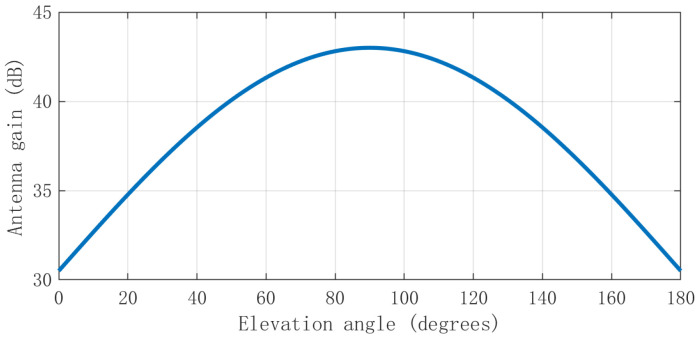
FRPAs gain graph [18].

The most widely used GNSS antenna is fixed reception pattern antennas (FRPAs). Figure 3 shows the typical FRPAs directional gain in commercial receivers. The relationship between the antenna gain and elevation angle is roughly a trigonometric one, so the antenna gain in a rotating antenna can be further simplified as [18]:

(3) $$G_{R}=f\left(\varphi ,\theta ,\beta ,\omega ,t\right)\approx u+g\cos\left(\omega t+\varphi_{0}-\varphi \right)+w$$

where *u* represents the constant component of the gain, *g* represents the varying component of the gain, and *w* represents Gaussian white noise.

The CNR of the navigation signal received by the receiver can be expressed as [17]:

(4) $$CNR=\frac{P_{R}}{N_{0}}=\frac{P_{T}G_{T}G_{R}\left(\theta ,\varphi \right)\lambda^{2}}{{\left(4\pi D\right)}^{2}N_{0}}$$

where $P_{R}$ represents the received power of the signal, $N_{0}$ represents the spectral power density of the noise, $P_{T}$ represents the transmission power of the signal, $G_{T}$ represents the gain of the transmitting antenna, $G_{R}\left(\theta ,\varphi \right)$ represents the gain of the receiving antenna when the incident elevation angle of the signal is φ, and the azimuth angle is θ, λ is the wavelength of the signal, and *D* is the distance between the transmitting antenna and the receiving antenna. If $p=P_{T}G_{T}\lambda^{2}/\left[{\left(4\pi D\right)}^{2}N_{0}\right]\;$, the received airborne noise ratio can be expressed as:

(5) $$CNR=G_{R}\left(\theta ,\varphi \right)p$$

Due to the distance between the navigation satellite and the ground receiver, λ, $N_{0}$, and *D* can be considered constant values in a short period, and the transmission power and gain of the navigation satellite remain unchanged, so *p* can be regarded as a constant value. Therefore, the change in antenna gain caused by rotating the antenna can be reflected through the CNR of the signal [17]. Bringing Formula (3) into (5) yields:

(6) $$CNR\approx pu+pg\cos\left(\omega t+\varphi_{0}+\varphi \right)+pw=D+A\cos\left(\omega t+\phi \right)+W=s\left(t;D,A,\phi \right)+W$$

where *D* is the constant component of the CNR, *A* is the varying component of the CNR, and *W* is the zero mean additive white Gaussian noise with variance $\sigma_{CNR}^{2}$. We represent the CNR measurement value as $x={\left[CNR\left(0\right),CNR\left(T_{0}\right),CNR\left(T_{1}\right),\ldots ,CNR\left(\left(N-1\right)T_{0}\right)\right]}^{T}$, where $T_{0}$ is the sampling interval. By using MLE to calculate the variables θ=[D,A,ϕ], a cost function is established as follows:

(7) $$J\left(\theta \right)=\sum_{n=0}^{N-1}{\left[CNR\left(nT_{0}\right)-D-Acos\left(\omega nT_{0}+\phi \right)\right]}^{2}=\sum_{n=0}^{N-1}{\left[CNR\left(nT_{0}\right)-D-Acos\left(\phi \right)cos\left(\omega nT_{0}\right)+Asin\left(\phi \right)sin\left(\omega nT_{0}\right)\right]}^{2}$$

According to the MLE, it can be obtained that $\hat{\phi }∼N\left(\phi ,\sigma_{\phi }^{2}\right)$ [18]:

(8) $$\hat{\phi }=\arctan\frac{-\sum_{n=0}^{N-1}CNR\left(nT_{0}\right)\sin\left(n\omega T_{0}\right)}{\sum_{n=0}^{N-1}CNR\left(nT_{0}\right)\cos\left(n\omega T_{0}\right)}$$

(9) $$\sigma_{\phi }^{2}=\frac{N\sigma_{CNR}^{2}}{2\left[{\left[\sum_{n=0}^{N-1}CNR\left(nT_{0}\right)cos\left(n\omega T_{0}\right)\right]}^{2}+{\left[\sum_{n=0}^{N-1}CNR\left(nT_{0}\right)sin\left(n\omega T_{0}\right)\right]}^{2}\right]}$$

Without considering multipath interference, for navigation signals from satellite *i* and satellite *j*, the CNR can be used to obtain the corresponding ${\hat{\phi }}_{i}∼N\left(\phi_{i},\sigma_{\phi ,i}^{2}\right)$ and ${\hat{\phi }}_{j}∼N\left(\phi_{j},\sigma_{\phi ,j}^{2}\right)$ of the two sets of signals. Assuming the azimuth angles of satellite *i* and satellite *j* relative to the rotating antenna are $\varphi_{i}$ and $\varphi_{j}$, then:

(10) $$d\phi_{ij}=\phi_{i}-\phi_{j}=\left(\varphi_{0}+\varphi_{i}\right)-\left(\varphi_{0}+\varphi_{j}\right)=\varphi_{i}-\varphi_{j}=d\varphi_{ij}$$

Therefore, $d{\hat{\phi }}_{ij}∼N\left(d\varphi_{ij},\sigma_{\phi ,i}^{2}+\sigma_{\phi ,j}^{2}\right)$, and it can be standardized as:

(11) $$\frac{d{\hat{\phi }}_{ij}-d\varphi_{ij}}{\sqrt{\sigma_{\phi ,i}^{2}+\sigma_{\phi ,j}^{2}}}∼N\left(0,1\right)$$

The above is the analysis result when the navigation signal is real. When there is a spoofing signal, the above formula will not conform to the standard normal distribution.

### 2.3. Parameter Estimation Based on Carrier Phase

The N phase measurements of the ith satellite received by the antenna during the rotation process at different times are:

(12) $$\Phi_{i}\left(k\right)=\rho_{i}\left(k\right)+\varepsilon_{i}\left(k\right),\;i=1,2,\ldots ,I;\;k=0,1,2,\ldots ,N-1$$

where $\rho_{i}\left(k\right)$ is the true carrier phase value of the ith satellite, and $\varepsilon_{i}\left(k\right)$ is the carrier phase measurement noise of the ith satellite. Due to the rotation of the antenna at angular velocity ω, $\rho_{i}\left(k\right)$ can be expressed as [16]:

(13) $$\rho_{i}\left(k\right)=\rho_{i,sat}\left(k\right)+r\cos\theta_{i}\cos\left(\omega T_{0}k+\varphi_{i}+\varphi_{0}\right)$$

where $\rho_{i,sat}\left(k\right)$ is the true value of the carrier phase at the stationary origin. Its change is mainly caused by the satellite’s motion relative to the origin. $\varphi_{i}$ and $\theta_{i}$ are the azimuth and elevation angles of the ith satellite’s signal incident into the coordinate system, and $\varphi_{0}$ is the initial rotation phase. *r* is the horizontal projection distance from the center of the antenna to the center of rotation, ω is the rotational angular velocity of the antenna, and $T_{0}$ is the sampling period of the receiver connected to the rotating antenna.

By performing forward and backward differentiation on the carrier phase measurement value in Formula (12), (14) can be obtained [16]:

(14) $$\begin{array}{c} d\Phi_{i}\left(k\right)=\Phi_{i}\left(k+1\right)-\Phi_{i}\left(k-1\right)\\ =\left\{\rho_{i,sat}\left(k+1\right)+r\cos\theta_{i}\cos\left[\omega T_{0}\left(k+1\right)+\varphi_{i}+\varphi_{0}\right]+\varepsilon_{i}\left(k+1\right)\right\}-\\ \left\{\rho_{i,sat}\left(k-1\right)+r\cos\theta_{i}\cos\left[\omega T_{0}\left(k-1\right)+\varphi_{i}+\varphi_{0}\right]+\varepsilon_{i}\left(k-1\right)\right\}\\ =d\rho_{i,sat}\left(k\right)-2r\sin\left(\omega T_{0}\right)\cos\theta_{i}\sin\left(\omega T_{0}k+\varphi_{i}+\varphi_{0}\right)+\gamma \left(k\right),k=1,2,\ldots ,N-2 \end{array}$$

where γ(k) is the new noise term obtained from the differential operation of the noise term.

For receivers in a stationary state, satellite navigation spoofing signals also need to simulate the changes in signal characteristics caused by navigation satellite motion; that is, the real signal and spoofing signal in Equation (14) have the same carrier phase change $d\rho_{i,sat}\left(k\right)$ caused by satellite motion, and the change in satellite motion can be calculated based on ephemeris data to obtain $d\rho_{i,sat}\left(k\right)$. Therefore, $d\rho_{i,sat}\left(k\right)$ can be used as a known quantity to obtain a differential sequence that only includes errors and carrier phase changes caused by the antenna rotation motion:

(15) $$d\Phi_{i,rcv}\left(k\right)=-2r\sin\left(\omega T_{0}\right)\cos\theta_{i}\sin\left(\omega T_{0}k+\varphi_{i}+\varphi_{0}\right)+W_{\Phi ,i}\left(k\right)$$

Continuing of the above equation, one can obtain the following:

(16) $$d\Phi_{rcv}\left(t\right)=M\sin\left(\omega t+\gamma \right)+W_{\Phi }=s\left(t;M,\gamma \right)+W_{\Phi }$$

where $M=-2r\sin\left(\omega T_{0}\right)\cos\theta_{i}$, $\gamma =\varphi_{1}+\varphi_{0}$, $W_{\Phi }$ is the zero mean additive white Gaussian noise with variance $\sigma_{\Phi }^{2}$. The differential value of the carrier phase measurement is represented as $x={\left[d\Phi_{rcv}\left(0\right),d\Phi_{rcv}\left(T_{0}\right),d\Phi_{rcv}\left(T_{1}\right),\ldots ,d\Phi_{rcv}\left(\left(N-1\right)T_{0}\right)\right]}^{T}$. The variable $\theta =\left[M,\gamma \right]$ is calculated through MLE and the cost function is established as follows:

(17) $$J\left(\theta \right)=\sum_{N-1}^{n=0}\;{\left[d\Phi_{rcv}\left(nT_{0}\right)-M\sin\left(\omega nT_{0}+\gamma \right)\right]}^{2}=\sum_{N-1}^{n=0}\;{\left[d\Phi_{rcv}\left(nT_{0}\right)-M\cos\left(\gamma \right)\sin\left(\omega nT_{0}\right)-M\sin\left(\gamma \right)\cos\left(\omega nT_{0}\right)\right]}^{2}$$

Based on MLE, $\hat{\gamma }∼N\left(\varphi +\varphi_{0},\sigma_{\gamma }^{2}\right)$ and $\hat{M}∼N\left(-2r\sin\left(\omega T_{0}\right)\cos\theta ,\sigma_{M}^{2}\right)$ can be obtained [18]:

(18) $$\hat{\gamma }=\arctan\frac{\sum_{N-1}^{n=0}\;d\Phi_{rcv}\left(nT_{0}\right)cos\left(n\omega T_{0}\right)}{\sum_{N-1}^{n=0}\;d\Phi_{rcv}\left(nT_{0}\right)sin\left(n\omega T_{0}\right)}$$

(19) $$\hat{M}=-\frac{2}{N}\sqrt{{\left[\sum_{N-1}^{n=0}\;d\Phi_{rcv}\left(nT_{0}\right)cos\left(n\omega T_{0}\right)\right]}^{2}+{\left[\sum_{N-1}^{n=0}\;d\Phi_{rcv}\left(nT_{0}\right)sin\left(n\omega T_{0}\right)\right]}^{2}}$$

(20) $$\sigma_{\gamma }^{2}=\frac{N\sigma_{\Phi }^{2}}{2\left[{\left[\sum_{N-1}^{n=0}\;d\Phi_{rcv}\left(nT_{0}\right)cos\left(n\omega T_{0}\right)\right]}^{2}+{\left[\sum_{N-1}^{n=0}\;d\Phi_{rcv}\left(nT_{0}\right)sin\left(n\omega T_{0}\right)\right]}^{2}\right]}$$

(21) $$\sigma_{M}^{2}=\frac{2\sigma_{\Phi }^{2}}{N}$$

Without considering multipath interference, for navigation signals from satellite *i* and satellite *j*, CPSD can be used to obtain the corresponding γ and *M* of two sets of signals, satisfying ${\hat{\gamma }}_{i}∼N\left(\varphi_{i}+\varphi_{0},\sigma_{\gamma ,i}^{2}\right)$,${\hat{\gamma }}_{j}∼N\left(\varphi_{j}+\varphi_{0},\sigma_{\gamma ,j}^{2}\right)$,${\hat{M}}_{i}∼N\left(-2r\sin\left(\omega T_{0}\right)\cos\theta_{i},\sigma_{M,i}^{2}\right)$ and ${\hat{M}}_{j}∼N\left(-2r\sin\left(\omega T_{0}\right)\cos\theta_{j},\sigma_{M,j}^{2}\right)$. Furthermore, $d{\hat{\gamma }}_{ij}∼N\left(d\varphi_{ij},\sigma_{\gamma ,i}^{2}+\sigma_{\gamma ,j}^{2}\right)$, standardized as:

(22) $$\frac{d{\hat{\gamma }}_{ij}-d\varphi_{ij}}{\sqrt{\sigma_{\gamma ,i}^{2}+\sigma_{\gamma ,j}^{2}}}∼N\left(0,1\right)$$

(23) $$\frac{{\hat{M}}_{i}+2r\sin\left(\omega T_{0}\right)\cos\theta_{i}}{\sigma_{M,i}}∼N\left(0,1\right)$$

(24) $$\frac{{\hat{M}}_{j}+2r\sin\left(\omega T_{0}\right)\cos\theta_{j}}{\sigma_{M,j}}∼N\left(0,1\right)$$

The above is the analysis result when the navigation signal is real. When there is a spoofing signal, the above variables will not conform to the standard normal distribution.

## 3. Spoofing Detection Method

By analyzing the impact of spoofing on parameters under a single rotating antenna, hypothesis testing is established based on observations of the CNR and carrier phase. Then, the GLRT method is used to solve the hypothesis-testing problem, thus achieving spoofing detection.

### 3.1. Hypothesis Test

First, the CNR and carrier phase of satellite *i* and satellite *j* are obtained by a single rotating antenna. Then, $d{\hat{\phi }}_{ij}$, $d{\hat{\gamma }}_{ij}$, ${\hat{M}}_{i}$, and ${\hat{M}}_{j}$ are calculated. Finally, the sum of squares due to error (SSE) is established to evaluate the differences between the estimated and predicted values of the above variables:

(25) $$SSE=\underset{SSE_{1}}{\underset{︸}{\frac{{\left(d{\hat{\phi }}_{ij}-d\varphi_{ij}\right)}^{2}}{\sigma_{\phi ,i}^{2}+\sigma_{\phi ,j}^{2}}}}+\underset{SSE_{2}}{\underset{︸}{\frac{{\left(d{\hat{\gamma }}_{ij}-d\varphi_{ij}\right)}^{2}}{\sigma_{\gamma ,i}^{2}+\sigma_{\gamma ,j}^{2}}}}+\underset{SSE_{3}}{\underset{︸}{\frac{{\left({\hat{M}}_{i}+2r\sin\left(\omega T_{0}\right)\cos\theta_{i}\right)}^{2}}{\sigma_{M,i}^{2}}+\frac{{\left({\hat{M}}_{j}+2r\sin\left(\omega T_{0}\right)\cos\theta_{j}\right)}^{2}}{\sigma_{M,j}^{2}}}}$$

where $SSE_{1}$ is determined by the azimuth angle estimated from the CNR, $SSE_{2}$ is determined by the azimuth angle estimated from the CPSD, and $SSE_{3}$ is determined by the elevation angle estimated from the CPSD.

If there is no spoofing signal, the estimated value of the above variables should be consistent with the predicted value, and the difference between the two should be a normal distribution of a zero mean value. In this case, the SSE statistical value should meet the Chi-squared distribution with four degrees of freedom, which is recorded as $\chi^{2}\left(4\right)$. When there is a spoofing signal, there will be a deviation between the estimated and predicted values of the above variables. The SSE should meet the non-central chi-squared distribution, with a degree of freedom of 4 and an eccentricity of Δ. Based on the above analysis, the following assumptions are established:

(26) $$\begin{array}{c} H_{0}:SSE∼\chi^{2}\left(4\right)\;no\;spoofing\\ H_{1}:SSE∼\chi^{2}\left(4,\Delta \right)\;spoofing\\ \Delta =\frac{{\left(d\phi_{ij}-d\varphi_{ij}\right)}^{2}}{\sigma_{\phi ,i}^{2}+\sigma_{\phi ,j}^{2}}+\frac{{\left(d\gamma_{ij}-d\varphi_{ij}\right)}^{2}}{\sigma_{\gamma ,i}^{2}+\sigma_{\gamma ,j}^{2}}+\frac{{\left(M_{i}+2r\sin\left(\omega T_{0}\right)\cos\theta_{i}\right)}^{2}}{\sigma_{M,i}^{2}}+\frac{{\left(M_{j}+2r\sin\left(\omega T_{0}\right)\cos\theta_{j}\right)}^{2}}{\sigma_{M,j}^{2}} \end{array}$$

The probability density function of the SSE under the zero hypothesis and alternative hypothesis is:

(27) p(SSE|H0)=SSEe−SSE−SSE224Γ(4)SSE≥0p(SSE|H1)=e−(SSE+Δ)−(SSE+Δ)22SSESSEΔΔ2I1(SSEΔ)SSE≥0

where Γ(⋅) is the gamma function and $I_{1}(\cdot )\;$ is the Bessel function of the first-order transformation of the first kind.

Establishing the GLRT:

(28) $$\lambda \left(SSE\right)=\frac{p(SSE|H_{1})}{p(SSE|H_{0})}\underset{H_{0}}{\overset{H_{1}}{≷}}\;\eta$$

where η is the detection threshold. According to the Neyman–Pearson criterion, the threshold value $SSE_{th}$ is set to determine the detection probability $P_{fa}$ under a specific false alarm probability $P_{D}$:

(29) $$\left\{\frac{P_{fa}=P\left\{SSE>SSE_{th}|H_{0}\right\}=1-\int_{0}^{SSEth}p_{\chi^{2}\left(4\right)}\left(SSE\right)dSSE}{P_{D}=P\left\{SSE>SSE_{th}|H_{1}\right\}=\int_{SSE_{th}}^{\infty }p_{\chi^{2}\left(4,\Delta \right)}\left(SSE\right)dSSE}\right.$$

**Figure 4:**
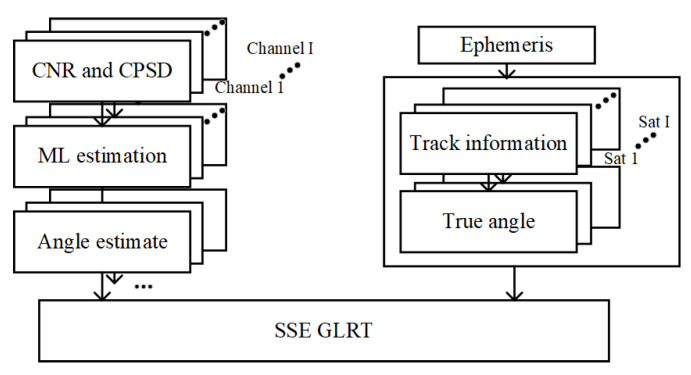
Architecture of spoofing-detection method.

Based on the above analysis, the spoofing-detection method shown in Figure 4 is designed, and the detailed process is as follows:

Phase 1: Data Collection

(1) Collect the CNR data of satellite *i*, defined as $CNR_{i}\left(k\right)$, where i=1,2,…,I represents the satellite number, and k=0,1,2,…,N−1 represents the time of the data.
(2) Collect the carrier phase measurement value of satellite *i*, defined as $\Phi_{i}\left(k\right)$. Calculate the carrier phase change value caused by the satellite’s motion relative to the origin O. Then, perform forward and backward differentiation on the carrier phase measurement value to obtain the CPSD, denoted as $d\Phi_{i}\left(k\right)$.

Phase 2: MLE of Parameters

(1) According to Formulas (8) and (9), calculate the estimated values and variances of the parameters ${\hat{\phi }}_{i}$ and ${\hat{\phi }}_{j}$ for the satellites *i* and *j*.
(2) Calculate the estimated values and variances of parameters ${\hat{\gamma }}_{i}$ and ${\hat{\gamma }}_{j}$ for satellites *i* and *j* according to Formulas (18) and (20), and calculate the estimated values and variances of parameters ${\hat{M}}_{i}$ and ${\hat{M}}_{j}$ for satellites *i* and *j* according to Formulas (19) and (21).
(3) Calculate the arrival angle difference $d\varphi_{ij}$ and elevation angles $\theta_{i}$ and $\theta_{j}$ for satellites *i* and *j* based on ephemeris information.

Phase 3: Likelihood Ratio Test

(1) Determine the monitoring threshold based on the set detection and false alarm probability.
(2) According to Formula (25), calculate the SSE. If the SSE is lower than the threshold, the signals of satellites *i* and *j* are real signals. Otherwise, at least one of the signals is a spoofing signal.

### 3.2. Performance Analysis

The receiver operator characteristic (ROC) can reflect the detection performance of the detection method when the threshold changes. This section takes the ROC as a reference and analyzes the factors affecting the detection performance through simulation. During the simulation, two navigation signals are set, with their true directions being (185°, 85°) and (270°, 65°). The spoofer simulates the transmission of the second set of navigation signals, and the angle at which the spoofing signal deviates from the true signal is represented as $\left(d\varphi_{2},\;d\theta_{2}\right)$. Assuming that the receiver is spoofed, the first set of signals it receives is a real signal, and the second set of signals is a spoofing signal with a different direction from the real signal. Unless otherwise specified, the parameter settings for the rotating antenna during the simulation process are shown in Table 1. The entire simulation process is based on Formulas (25)–(29). By inputting the parameters from Table 1 into Formula (29) to calculate the ROC curve, the relationship between relevant factors and detection performance can be further analyzed. During this process, no navigation signal is generated and no navigation solution is calculated.

**Figure 5:**
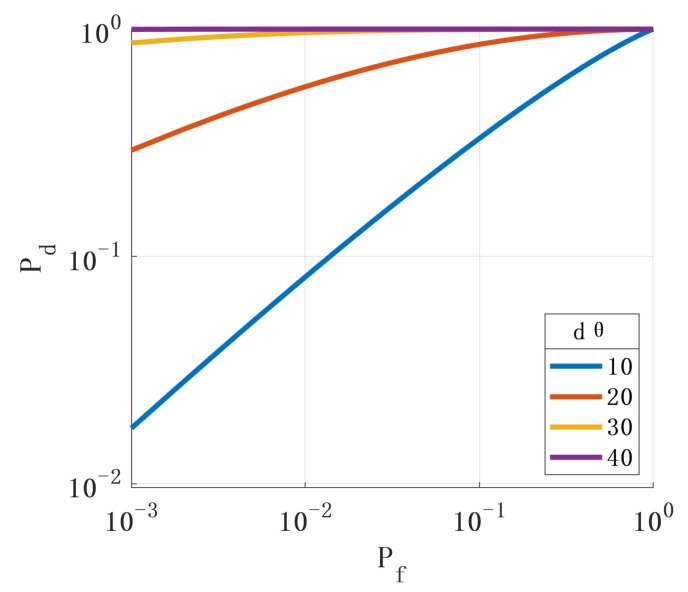
Relationship between the deviation of the spoofing signal pitch angle from the true signal angle and ROC.

**Figure 6:**
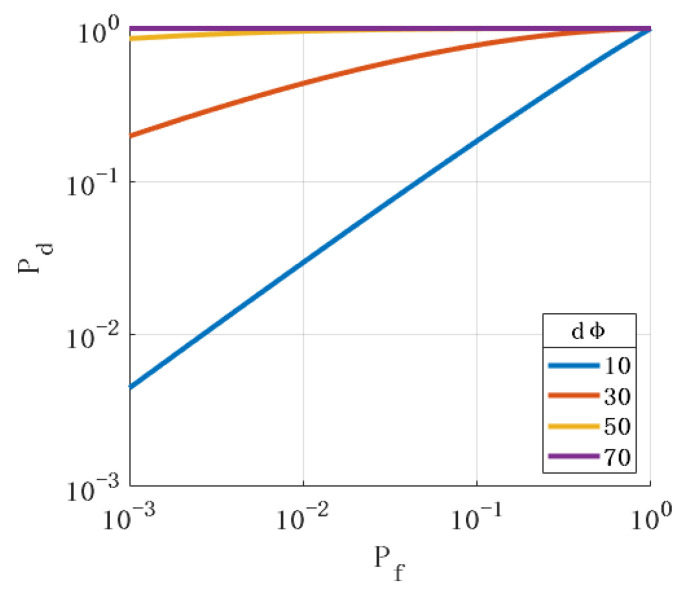
Relationship between the deviation of the spoofing signal azimuth angle from the true signal angle and ROC.

First, we analyzed the impact of the spoofing signal deviation angle on the performance of the spoofing-detection methods. Figure 5 shows the ROC when the spoofing signal’s pitch angle direction deviates from the real signal’s angle. Figure 6 shows the ROC when the azimuth direction of the spoofing signal deviates from the angle of the real signal. The simulation results show that the more the incident angle of the spoofing signal deviates from the true signal, the better the detection performance.

**Figure 7:**
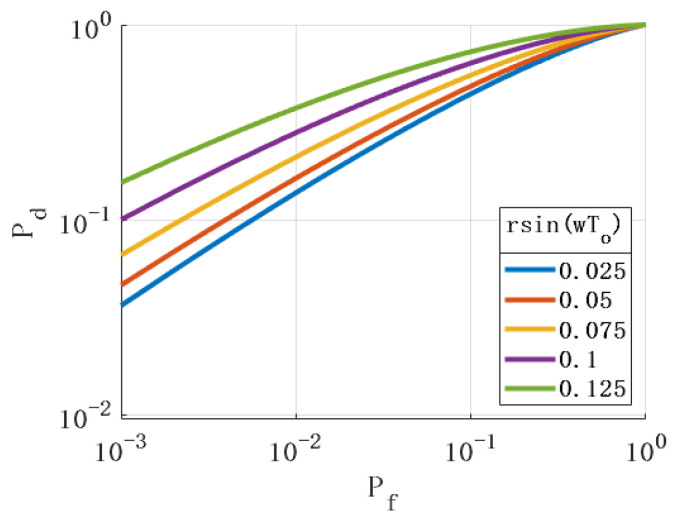
Relationship between $r\sin(\omega T_{0})$ and ROC.

**Figure 8:**
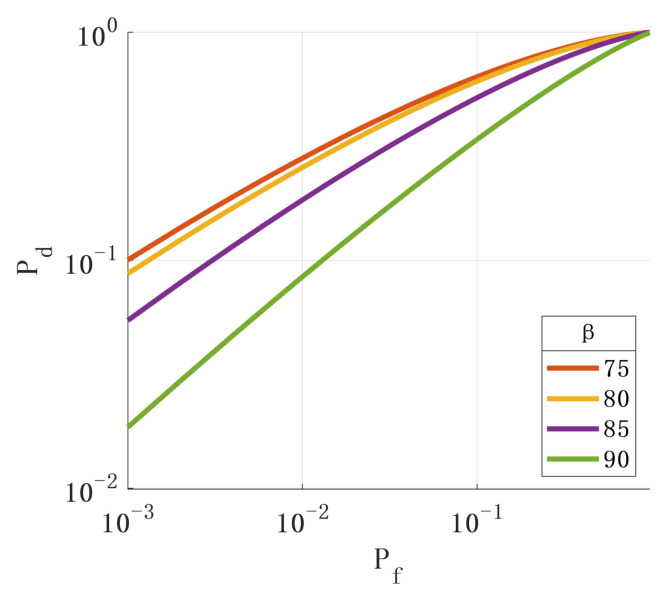
Relationship between β and ROC.

**Figure 9:**
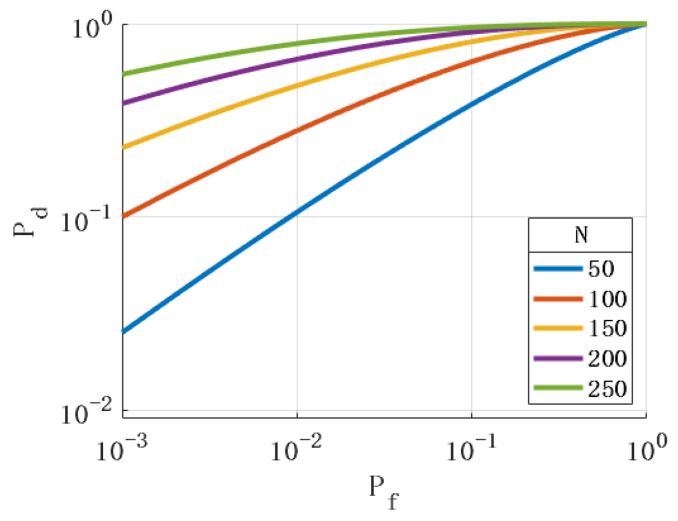
Relationship between *N* and ROC.

By observing Formula (26), it was found that the parameters $r\sin(\omega T_{0})$, β, and *N* also affect the performance of the detection algorithm. Figure 7, Figure 8 and Figure 9 show the ROC with different values of the above parameters. The simulation results show that the changes in the above parameters will affect the ROC. By analyzing the simulation results, the following conclusions can be drawn: (1) The larger the angle between the axis of the rotating antenna and the horizontal plane, the better the spoofing detection performance. (2) The more sampled data, the better the performance of the spoofing detection. (3) The larger the horizontal projection distance from the antenna center to the rotation center, the better the detection performance. The horizontal projection distance is the product of the cosine value of the antenna inclination angle and the radius. When the inclination angle of the antenna is determined, the larger the antenna radius, the better the detection performance. (4) The closer the product of the antenna rotation angular velocity and sampling period is to π, the better the detection performance.

**Figure 10:**
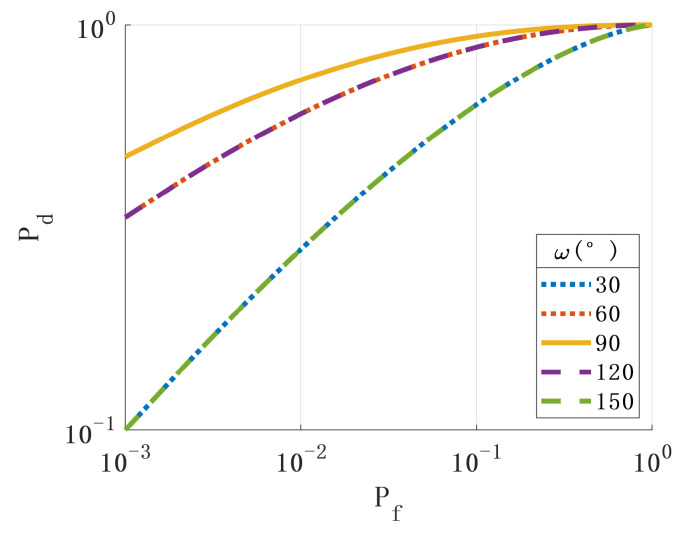
Relationship between ω and ROC.

Figure 10 shows the relationship between the rotation speed and the detection performance. It can be seen that the detection performance is proportional to $\sin(\omega T_{0})$, which is consistent with the simulation results in Figure 7.

In addition, the detection performance will decrease with the increase of noise variance, and the gain map of the antenna will also affect the detection performance, but it is not significant.

## 4. Experimental Results

To verify the effectiveness of the proposed method and evaluate its performance, a spoofing environment was set up for experimental verification.

### 4.1. Environment Setup and Parameter Setting

**Figure 11:**
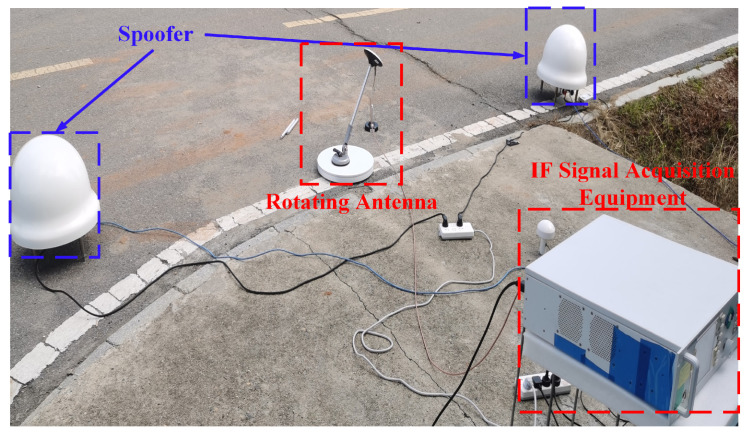
Spoofing scenario.

Firstly, the spoofing scenario constructed is introduced. Figure 11 shows a spoofing scenario consisting of two spoofing sources, a single rotating antenna, and a piece of intermediate frequency signal-acquisition equipment. Two spoofing sources simulate multi-agent spoofing devices sending spoofing signals, and the intermediate frequency signal collection equipment is connected to a single rotating antenna to store the navigation signals received by the single rotating antenna. Two spoofers are connected to the same terminal. The terminal controls two spoofers to send a total of six spoofing signals to achieve time spoofing. Subsequently, through the FGI-GSRx software receiver (the codes can be downloaded from https://github.com/nlsfi/FGI-GSRx, accessed on 5 September 2023), we processed the intermediate frequency signal in the dataset and validated the spoofing detection algorithm on the processed data.

The configuration and parameter settings of the experiment are shown in Table 2. The single rotating antenna was placed at the position of (0,0,0), and spoofers A and B were located at (0,2,0.5) and (0,−2,0.5) in the local coordinate system, with the coordinates in meters.

**Figure 12:**
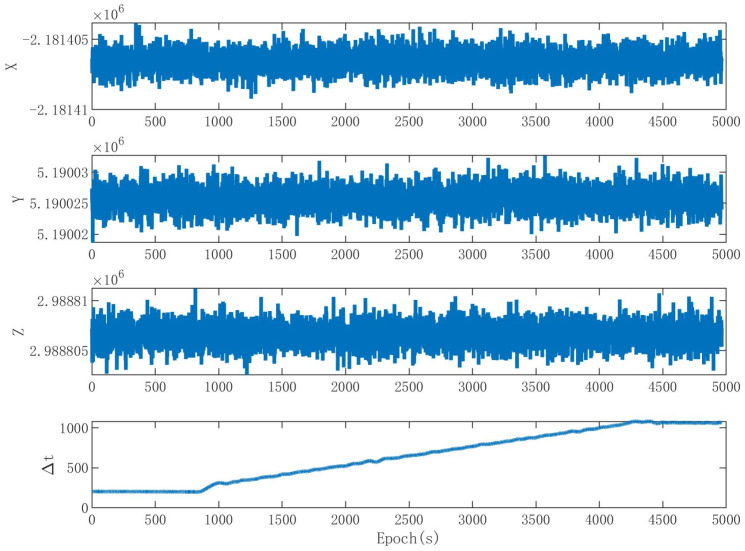
Navigation solution in the experiment.

Figure 12 shows the navigation solution throughout the entire spoofing process. The position information in the navigation solution remained stable. The time information in the navigation solution is consistent with the spoofing strategy.

### 4.2. Method Validation and Performance Analysis

**Figure 13:**
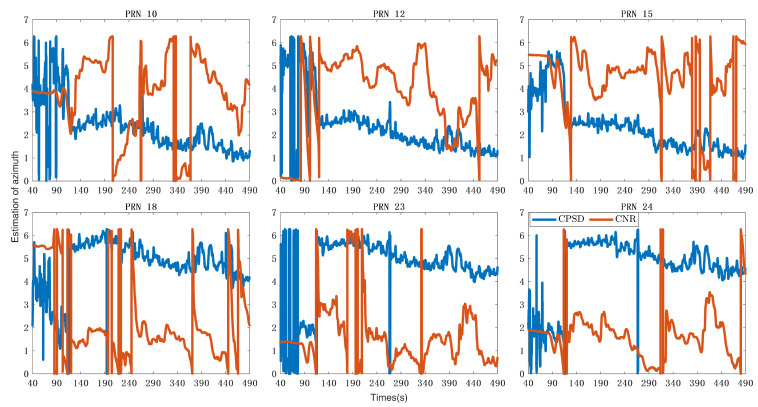
Azimuth estimation value calculated based on CNR and CPSD. The unit of ordinate is radians.

Figure 13 shows the estimation of azimuth obtained from CNR and CPSD estimation. It can be seen that there is a high similarity between the estimation of spoofing from the same direction. Due to differences in MLE algorithms, the initial phase of the two estimated values is different. Therefore, there is a difference between the two estimated values in the figure. When calculating the detection variables, the initial phase will be eliminated by difference.

**Figure 14:**
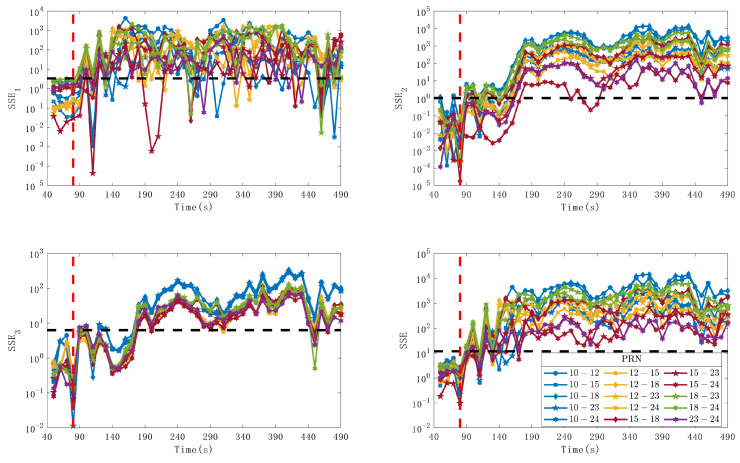
The variation curves of detector values under different satellite combinations. The colors of the lines and patterns of the points represent different satellites. Blue indicates that the combination contains PRN10, yellow and round indicates that the combination contains PRN12, red and square indicates that the combination contains PRN15, green and diamond indicates that the combination contains PRN18, purple and pentagram indicates that the combination contains PRN23, and a hexagon indicates that the combination contains PRN24. The black dashed line represents the optimal detection threshold calculated based on the Neyman–Pearson criterion, and the red dashed line represents the time of the spoofing start.

**Figure 15:**
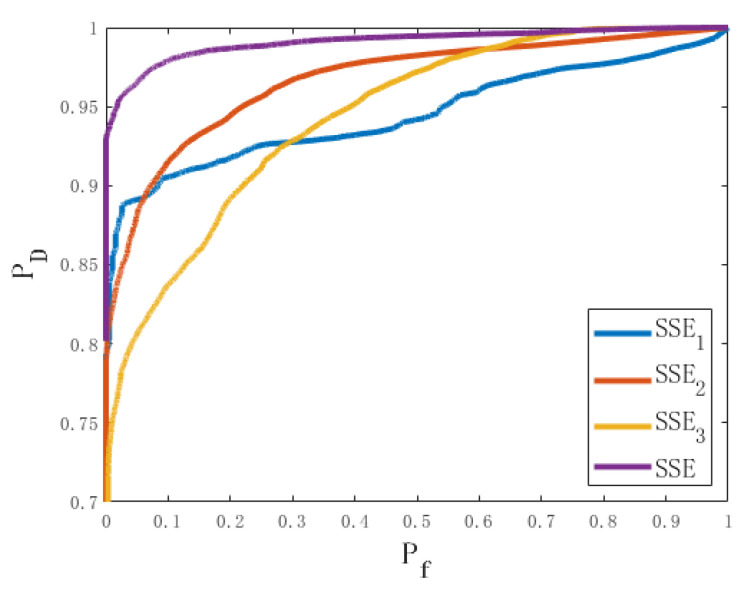
ROC curves under different detection variables.

After processing the data in the spoofing experiment, the spoofing-detection method proposed in this article was verified and its performance was analyzed. To analyze the impact of the components of the detection variable SSE on detection performance, the detection performance of $SSE_{1}$, $SSE_{2}$, $SSE_{3}$, and SSE was evaluated. $SSE_{1}$ is determined by the azimuth angle estimated from the CNR, $SSE_{2}$ is determined by the azimuth angle estimated from the CPSD, and $SSE_{3}$ is determined by the elevation angle estimated from the CPSD. Figure 14 shows the variation of detection variables under different satellite combinations. To facilitate observation, the detection variables of each pair of combinations are distinguished by color and pattern. The satellite PRNs represented by different colors and patterns are described in the caption. The black dashed line in the figure indicates the optimal detection threshold calculated based on the Neyman–Pearson criterion. The value of the detection variable that is greater than the threshold indicates that the pair of signals contains a spoofing signal. It should be noted that 40 s of data are required for parameter estimation using MLE, so the length of the detection variable is 450 s. By selecting different thresholds and obtaining the detection probability and false alarm probability under different thresholds, the ROC shown in Figure 15 is obtained on this basis.

The optimal detection probability and optimal false alarm probability of $SSE_{1}$, $SSE_{2}$, $SSE_{3}$, and SSE in the experimental data were calculated according to the Neyman–Pearson criterion, as shown in Table 3.

By observing Figure 14, it is found that: (1) $SSE_{1}$ has good detection performance in the initial stage of spoofing but has a high probability of missed detection during the continuous spoofing process; (2) $SSE_{2}$ and $SSE_{3}$ have poor detection performance in the initial stage of spoofing, but have good detection performance during the continuous spoofing process; (3) SSE has good detection performance in both the initial spoofing stage and the continuous spoofing stage. By observing Figure 15 and Table 3, it is found that the detection performances of $SSE_{1}$ and $SSE_{2}$ are close to each other and higher than that of $SSE_{3}$, and the performance of the spoofing detection using the elevation angle is slightly lower than that using the azimuth angle. The detection performance of SSE is significantly better than that of $SSE_{1}$, $SSE_{2}$, and $SSE_{3}$, indicating that using both CNR and CPSD for spoofing detection has better detection performance. The values of different combinations of detection variables are above the detection threshold, indicating that the method has good immediacy. By calculating the average response time of the detection variable SSE to first exceed the detection threshold after spoofing occurs, it is found that the average response time is approximately 3.2 s, indicating that the method has good immediacy.

In summary, for navigation signals in pairs, using MLE for parameter estimation and comparing the detection variable with the threshold can determine whether the combination contains spoofing signals. The above experimental results demonstrate the effectiveness and immediacy of this method.

## 5. Conclusions

This article proposes a spoofing-detection method based on angle comparison applied to a single rotating antenna. Based on MLE, the estimated value of IA-DOA is calculated using the CNR and CPSD of multiple epochs. Detection variables are established based on the predicted and estimated values of the IA-DOA. The influence of the antenna’s parameters on the detection performance was analyzed through simulation. Finally, on-site experiments were conducted to validate and evaluate the proposed method. The results indicate that the method proposed in this paper can effectively achieve real-time detection of multi-agent spoofing. This method can be applied to spoofing detection for fixed station receivers.

Compared with other spoofing-detection methods based on signal spatial correlation, this method only requires a single rotating antenna to achieve multi-agent spoofing detection. Compared with other detection methods based on a single rotating antenna, this method not only has higher detection performance but also achieves the detection of multi-agent spoofing.

## Figures and Tables

**Table 1:** Simulation parameter settings.

Parameter	Value
r(m)	0.2
$\omega {(}^{{}^{\circ}}/s)$	30
$T_{0}\left(s\right)$	1
$\beta {(}^{{}^{\circ}})$	70
*N*	100
$\sigma_{CNR}^{2}\left(dB^{2}Hz^{2}\right)$	1
$\sigma_{\Phi }^{2}\left(m^{2}\right)$	0.02
$d\varphi_{2}{(}^{{}^{\circ}})$	10
$d\theta_{2}{(}^{{}^{\circ}})$	10

**Table 2:** Parameter settings of the experiment.

Parameter setting of the receiver			
Rotating radius		0.15 m	
Tilt angle		30°	
Rotational angular velocity		18 °/s	
Sampling frequency		10 Hz	
Position coordinates		(0,0,0)	
Number of samples in MLE		400	
Parameter setting of spoofers			
Spoofer A		Spoofer B	
Position coordinates	(0,2,0.5)	Position coordinates	(0,−2,0.5)
PRN of spoofing	10,12,15	PRN of spoofing	18,23,24
Parameter setting of spoofing			
Start time of experiment		2023/09/11 06:12:05 (UTC)	
Experiment duration		490 s	
Start time of spoofing		80th second	
Type of spoofing		Time spoofing	

**Table 3:** Optimal detection probability and false alarm probability under different detection variables.

Detection Variables	$\mathrm{SS}E_{1}$	$\mathrm{SS}E_{2}$	$\mathrm{SS}E_{3}$	SSE
Optimal detection probability	0.8874	0.8853	0.7917	0.9545
Optimal false alarm probability	0.0258	0.0523	0.0321	0.0206

## Data Availability

The datasets generated during this study are available from the corresponding author upon request.

## Abbreviations

The following abbreviations are used in this manuscript:

GNSS	Global Navigation Satellite System
IA-DOA	intersection angle between two directions of arrival
CNR	carrier-to-noise ratio
CPSD	carrier phase single difference
GLRT	generalized likelihood ratio testing
